# Endoscopic sleeve gastroplasty: Prospective assessment of weight loss and metabolic impact

**DOI:** 10.1055/a-2819-0356

**Published:** 2026-03-17

**Authors:** Marc Barthet, Geoffroy Vanbiervliet, Jean-Michel Gonzalez, Maxime Thobois, Yoann Poher, Marion Blin, Shani Diai, Nathalie Lesavre, Sandrine Boullu

**Affiliations:** 1 37059 Gastroenterology, Hôpital Nord, Marseille, France; 2 Pôle digestif, Hôpital de L'Archet 2, Nice, France; 3 37045 Gastroenterology, CHU Nice, Nice, France; 4 37059 Hôpital Nord, Marseille, France; 5 90255 Aix-Marseille Université Faculté de Médecine, Marseille, France

**Keywords:** Endoscopy Upper GI Tract, Gastrostomy and PEJ, Dilation, injection, stenting, GI Pathology

## Abstract

**Background and study aims:**

Recent international guidelines (American Society for Gastrointestinal Endoscopy and European Society of Gastrointestinal Endoscopy) suggest endoscopic treatment plus lifestyle management for management of obesity class I and II. The aim of this prospective study was to assess results of Endosleeve gastroplasty (ESG) plus lifestyle management (LSM).

**Patients and methods:**

Twenty-nine patients (mean age 44 years) were included in a longitudinal prospective cohort study in two experienced centers. Mean weight at inclusion was 91.13 kg (± 8.4) and mean body mass index (BMI) was 34.08 (± 2.43) kg/m ^2^ . ESG was performed using Endomina (Endo Tools Therapeutics S.A., Gosselies, Belgium) 6 months after LSM and patients were followed for 1 year with continuous LSM.

**Results:**

Twenty-eight patients completed 12-month follow-up. Total body weight loss (TBWL) was 11.05% ( *P* < 0.001, 81.2% of cases with TBWL decrease > 5 %). EWL was significantly reached in 40.98% ( *P* < 0.001). Final BMI was significantly decreased (30.52 kg/m ^2^ ; *P* < 0.001). Glucose control and HbA1c level were significantly improved at 1 year ( *P* = 0.01 and *P* = 0.003). Liver function tests (gamma-glutamyl transferase, alanine aminotransferase) were significantly decreased ( *P* = 0.006 and *P* = 0.009). Diastolic arterial pressure significantly decreased at 1 year ( *P* < 0.001). Quality of life (QoL) score (sf12) for physical activity was significantly improved ( *P* = 0.05). Only one adverse event was noted (sustained pain; AGREE II). No nutritional deficiency assessed by biological measurements appeared during follow-up.

**Conclusions:**

ESG combined with LSM resulted in significant improvement in TBWL, EWL, BMI, and QoL. Metabolic improvements were also observed without leading to nutritional deficiency.

## Introduction

Obesity is a global pandemic, with a 2-fold increase in prevalence since 1980 1 . Overweight and obesity are now the fifth most common cause of mortality according to World Health Organization data 2 . In addition, obesity is associated with a panel of chronic diseases such as cardiovascular disease, diabetes (mainly type 2), metabolic-associated liver disease (MASH), sleep apnea, arthrosis, and cancers 1 2 3 . Type 2 diabetes and cardiovascular disease have been linked to obesity in 44% and 23% of cases, respectively, and an estimated 7% to 41% of cancers 1 2 3 4 . It has been demonstrated that excess weight increases both morbidity and mortality, with a significant increase at a body mass index (BMI) threshold of ≥ 28 kg/m ^2^ 1 4 . At this threshold, life expectancy drops by 7.1 years in women and 5.8 years in non-smoking men 2 .

Surgical procedures such as sleeve gastrectomy and bariatric bypass are effective but are restricted in most Western countries to patients with BMI ≥ 40 kg/m ^2^ or ranging between 35 kg/m ^2^ and 40 kg/m ^2^ without comorbidity 5 . Two recent randomized controlled trials (RCTs) with a crossover design showed that the percentage of total body weight loss (TBWL) with lifestyle management (LSM) alone ranged from 0% to 3% 6 7 .

Therefore, endoscopic bariatric and metabolic therapies (EBMTs) have been developed, including intragastric balloon (IGB) and endoscopic sleeve gastroplasty (ESG) 5 . ESG was associated with a TBWL rate of 10.8% to 13.8% in two RCTs 6 7 . In a large meta-analysis and systematic review that included 2,170 patients with a mean BMI ≥ 35.7 kg/m ^2^ , TBWL associated with ESG at 1-year follow-up was 16.1% and was 16.8% at 18 months 8 . Based on these results, American Society for Gastrointestinal Endoscopy (ASGE)/European Society of Gastrointestinal Endoscopy (ESGE) guidelines suggest use of EBMT with LSM in patients over LSM alone for those with BMI ≥ 30 kg/m ^2^ or BMI ≥ 27 kg/m ^2^ with at least one comorbidity 5 .

Despite one RCT and the crossover prospective study, various meta-analyses, and international guidelines, patients do not yet have access to reimbursement in most Western countries for obesity class I or class II without comorbidity, thus complicating the management of these patients with obesity. Therefore, this study assessed the efficacy of ESG combined with LSM in a longitudinal prospective cohort study including patients with BMI ≥ 30 kg/m ^2^ and BMI < 35 kg/m ^2^ (Class I) with or without comorbidity or BMI ≥ 35 kg/m ^2^ and < 40 kg/m ^2^ (Class II) without comorbidity, these indications being complementary to bariatric surgical indications and required by French National Health Insurance. The primary objective was weight loss in terms of TBWL; secondary objectives were excess weight loss (EWL) and BMI, metabolic assessment (e.g., glucose control, liver function tests, arterial pressure), safety, and quality of life (QoL).

## Patients and methods

### Patients

Twenty-nine patients (mean age 44 years) were prospectively included between 2019 and 2022 in two academic centers (Marseille, Nice), experienced and dedicated to management of obesity. Inclusion criteria were patients with BMI ≥ 30 kg/m ^2^ and BMI < 35 kg/m ^2^ (Class I) with or without comorbidity or those with BMI ≥ 35 kg/m ^2^ and < 40 kg/m ^2^ (Class II) without comorbidity. The study was approved by the local ethics committee and registered in the ClinicalTrials.gov database (NCT03900481). Patients eligible for the study based on their medical history, physical, psychological, and dietary examinations were included upon signing informed consent.

Prior to ESG eligibility, patients underwent 6 months of LSM under the supervision of a dedicated team including endocrinologists, dietitians, psychologists, and gastroenterologists. Patients were then followed for 1 year with scheduled appointments at 1 month, 3 months, 6 months, and 1 year.

The final included patients were: 1) those with BMI ≥ 30 kg/m ^2^ and BMI < 35 kg/m ^2^ (Class I) with or without comorbidity or BMI ≥ 35 kg/m ^2^ and < 40 kg/m ^2^ (Class II) without comorbidity; 2) and who underwent 6 months of LSM under the supervision of a dedicated team; 3) and who underwent 1 year of follow-up at 1, 3, and 6 months and 1 year.

ESG was performed using the Endomina device (Endo Tools Therapeutics, Gosselies, Belgium) after multidisciplinary discussion and after obtaining informed consent. Patients stayed one night in the hospital after the procedure. After the procedure and 1 day of fasting, patients received a liquid diet for 1 week, then mashed food the following week, and then a soft diet during follow-up under dietitian supervision. The SF-12 quality of life score (Medical Outcomes Study Short-Form Health Survey) was assessed at inclusion and during follow-up.

### Endpoints

ESG response (primary endpoint) was the percentage of patients who had a TBWL > 5% at 12-month follow-up, compared with baseline and as suggested by recent international guidelines 5 . Secondary endpoints were TBWL, EWL, BMI, BMI classes (overweight, class I, class II), metabolic outcomes (glucose regulation (glycemia, HbA1c), triglycerides, liver function (aspartate aminotransferase [ASAT], alanine aminotransferase [ALAT], gamma-glutamyl transpeptidase [GGT], alkaline phosphatase [ALP), arterial pressure, ferritin), adverse events (AEs) (anemia, or changes in prealbumin, or vitamin D, vitamin A, vitamin B9, vitamin B12 level deficiencies), and QoL (SF-12 QoL score).

### Endomina procedure

Endosleeve gastroplasty procedure with one suture and the final result after 7 sutures.Video 1

Endomina is a single-use triangulation platform. It is inserted over two guidewires and assembled within the stomach with a forward-seeing endoscope with a 2.8-mm working channel. Endomina can accommodate 5F preloaded needles called TAPES (Transmural Antero-Posterior Endoscopic Suture) inside a flexible arm, giving additional degrees of freedom to the endoscope.

Endomina and TAPES were used to suture the stomach, starting from the anterior wall to the posterior wall, grasping the gastric wall with forceps (Raptor, US Endoscopy, Ohio, United States). The number of sutures made was between 6 and 8, starting upstream of the angulus ( Video 1 ).

### Statistical analysis.

Baseline characteristics are reported for the entire study population. Qualitative variables are expressed as numbers and percents, whereas quantitative variables are reported as medians (interquartile range). Normality of distribution was assessed using the Shapiro-Wilk test and visual inspection of histograms.

All tests were two-sided, with *P* < 0.05 considered statistically significant. Statistical analyses were performed using IBM SPSS Statistics, version 20.0.0 (IBM Corp., Armonk, New York, United States)

## Results

### Patient characteristics

Twenty-nine patients were included, but one was lost to follow-up (during the COVID lockdown). Therefore, 28 patients completed 12-month follow-up. All 28 patients were women with a mean age of 44.08 (11.21) years and 22 were smokers (78.6%). Sixteen patients with obesity class I had comorbidities (57.1%) including 14 patients with arterial hypertension and two with type 2 diabetes. Patients belonging to obesity class II had no comorbidities.

Mean weight at inclusion was 91.13 kg (8.4) and mean BMI was 34.08 kg/m ^2^ (2.43), including 20 patients with class I and nine with class II obesity. Mean systolic arterial pressure among included patients was 123.5 mm Hg (17.06) (maximum 169) and the diastolic arterial pressure was 71.43 mm Hg (8.41) (maximum 90). Mean level of glycated Hb (HbA1c) was 5.59 (0.33%) and mean triglyceride plasma level was 1.20 g/L (0.74) (maximum 3.5 g/L). Liver function tests were performed: ASAT 23.27 IU/L (8.14) (75th percentile 26.25); ALAT 24.73 IU/L (17.47) (75th percentile 26.25); GGT 28.5 IU/L (20.19) (75th percentile 30); and ALP 77.74 IU/L (23.96) (75th percentile 85).

### Weight loss outcomes

**Fig. 1:**
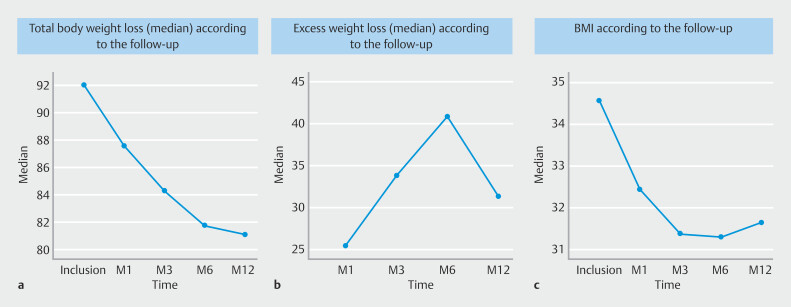
Weight loss outcomes according to follow-up. **a** Total body weight loss (TBWL) (kg). **b** Excess weight loss (EWL) %. **c** Body mass index (BMI) (kg/m2).

Weight loss outcomes are shown in Table 1 . For the primary endpoint of ESG response, the responder rate (TBWL > 5%) at 12-month follow-up was 23 (82.1%) (95% confidence interval [CI] 66%-96%) ( Fig. 1 **a** ). For secondary endpoints, responder rates at 1, 3, and 6 months post-inclusion were 19 (67.9%) (95% CI 51%-85%), 23 (82.1%) (95% CI 59%-100%), and 23 (82.2%) (95% CI 68%-97%), respectively. In addition, at 1-year follow-up, there was a significant decrease in TBWL of 11.05% (7.1) compared with baseline. Minimum weight loss was 5.6 kg and maximum weight loss was 23.2 kg. A total of 82.1% of patients were responders with TBWL > 5% (half of these with TBWL > 10%), and 17.9% were non-responders (TBWL < 5%). The TBWL rates over time at 1, 3, 6, and 12 months of follow-up were 6.01%, 9.05%, 11.13%, and 11.05%, respectively.

**Table:** **Table 1** Weight loss outcomes.

	**Inclusion**	**1 month**	**3 months**	**6 months**	**12 months**	***P***
Primary endpoint						
TBWL response > 5 (% and 95%CI)	NA	19 (67.9)	23 (82.1)	23 (82.1)	23 (82.1)	< 0.001
Secondary endpoints						
TBWL (%)	NA	6.01	9.05	11.13	11.05	< 0.001
EWL (%)	NA	23.69	34.79	41.61	40.98	< 0.001
BMI value (kg/m2): median (IQR)	33.99 (32.15–35.29)	31.91 (29.78–33.48)	31.04 (29.04–32.65)	31.24 (28.66–32.25)	31.66 (28.65–32.48)	< 0.001
BMI class						< 0.001
Class I	19 (67.90%)	15 (53.60%)	16 (57.10%)	17 (63%)	16 (64%)	
Class II	9 (32.10%)	5 (17.80%)	2 (7.10%)	0	0	
Overweight	0	8 (28.60%)	10 (35.80%)	10 (37%)	9 (36%)	
BMI, body mass index; CI, confidence interval; EWL, excess weight loss; IQR, interquartile range; TBWL, total body weight loss.						

EWL significantly increased over time, reaching 40.98% up to 1 year (min -31.88, max 113.02%) ( Fig. 1 **b** ). EWL at 1-, 3-, 6-, and 12-month follow-up was, 23.69%, 34.79%, 41.61%, and 40.98%, respectively.

Final BMI was 30.52 kg/m ^2^ (2.92) (34.08 kg/m ^2^ [2.43] at inclusion) with an overall significant decrease in BMI, resulting in complete absence of patients with class II obesity compared with baseline ( Fig. 1 **c** ). At the end of the study, there were no patients with class II obesity, 16 patients (64%) had BMI 30 to 35 kg/m ^2^ , nine patients (36%) had BMI values ranging between 25 and 30 kg/m ^2^ . Nadir was reached at 3 months for BMI and 6 months for weight loss. BMI values over time at 0, 1, 3, 6, and 12 months of follow-up were 32.03 kg/m ^2^ (2.39); 30.98 kg/m ^2^ (2.30); 30.37 kg/m ^2^ (2.46); and 30.52 kg/m ^2^ (2.92), respectively. BMI classes over time were: overweight n = 0, n = 8, n = 10, n = 10, n = 9; Class I n = 20, n = 15, n = 17, n = 16; Class II n = 8, n = 5, n = 2, n = 0, n = 0 at 1, 3, 6, and 12 months of follow-up, respectively.

### Metabolic outcomes

Data for metabolic analyses conducted at baseline and 12 months are shown in Table 2 .

**Table:** **Table 2** Metabolic outcomes.

	**Inclusion**	**12 months**	*P* value ^*^
	**m (IQR)**	**m (IQR)**	
Glycemia (mmol/L)	**5.30 (4.80–5.60)**	**5.00 (4.70–5.30)**	**0.011**
HbA1c (%)	**5.60 (5.37–5.82)**	**5.50 (5.37–5.70)**	**0.003**
Triglyceride (mmol/L)	0.90 (0.80–1.40)	0.90 (0.65–1.45)	0.275
Liver function test (IU/L)			
ASAT	21.50 (18.75–23.00)	21.00 (19.00–26.00)	0.891
ALAT	**20.00 (15.50–26.25)**	**18.00 (13.00–24.00)**	**0.006**
GGT	**22.50 (18.00–30.00)**	**17.00 (14.00–30.00)**	**0.009**
ALP	71.00 (63.50–85.00)	67.00 (60.00–87.00)	0.211
Arterial pressure (mmHg)			
Systolic	**123.50 (113.50–141.75)**	**122.00 (112.00–129.00)**	**0.016**
Diastolic	70.00 (63.25–79.25)	68.00 (63.00–68.00)	0.132
Ferritin (µg/L)	88.30 (44.20–142.07)	65.00 (36.50–135.60)	0.382
^*^P value for paired-sample t-tests or Wilcoxon tests. ASAT, aspartate aminotransferase; ALAT, alanine aminotransferase; ALP, alkaline phosphatase; GGT, gamma-glutamyl transpeptidase; m (IQR), median (interquartile range).			

#### Glycemia and triglycerides

There were significant decreases in glycemia levels (4.99 (0.5) vs 5.25 (0.64) mmol/L) and HbA1c (5.50 [0.39%] vs 5.59 [0.33%]) over the study period. There was no significant change in triglyceride plasma levels (1.20 (0.74) vs 1.09 (0.62) mmol/L).

#### Liver function tests

There was no change in ASAT (22.78 IU/L (5.54) vs 23.27 IU/L [8.14]) or ALP (74.85 IU/L [21.81] vs 77.74 IU/L [23.96]) values during the study period. A significant decrease was observed for ALAT (19.56 IU/L [8.58] vs 24.73 [17.47]) and GGT (23.37 IU/L 9 vs 28.5 IU/L [20.1]) values between baseline and 12 months.

#### Arterial pressure

There was a significant improvement in systolic arterial pressure at 6 months (127.54 mm Hg [15.61] vs 120.56 mm Hg [17.06]), but this was not significant at 1 year. There was a significant improvement at 1 year for diastolic arterial pressure (71.43 mm Hg [8.41] vs 69.54 mm Hg [9.01]).

### Safety

One AE occurred with sustained pain leading to a 2-day increase in hospital stay (AGREE II). Nutritional deficiencies were monitored through biological analyses at baseline and at the end of follow-up and no significant deficiencies were observed regarding anemia (Hb 13.37 g/dL [1.08] vs 13.16 g/dL [1.07]), prealbumin (0.25 g/L [0.05] vs 0.24 g/L [0.06]), vitamin D (76.04 ng/mL [25.79] vs 85.49 ng/mL [28.04]); vitamin A (0.60 ng/mL [0.21] vs 0.58 ng/mL [0.2]); vitamin B9 (18.5 ng/mL [4.8] vs 20.52 ng/mL [12.5]), and vitamin B12 (0.30 ng/mL [0.10] vs 0.31 ng/mL [0.10]).

### Quality of life

**Fig. 2:**
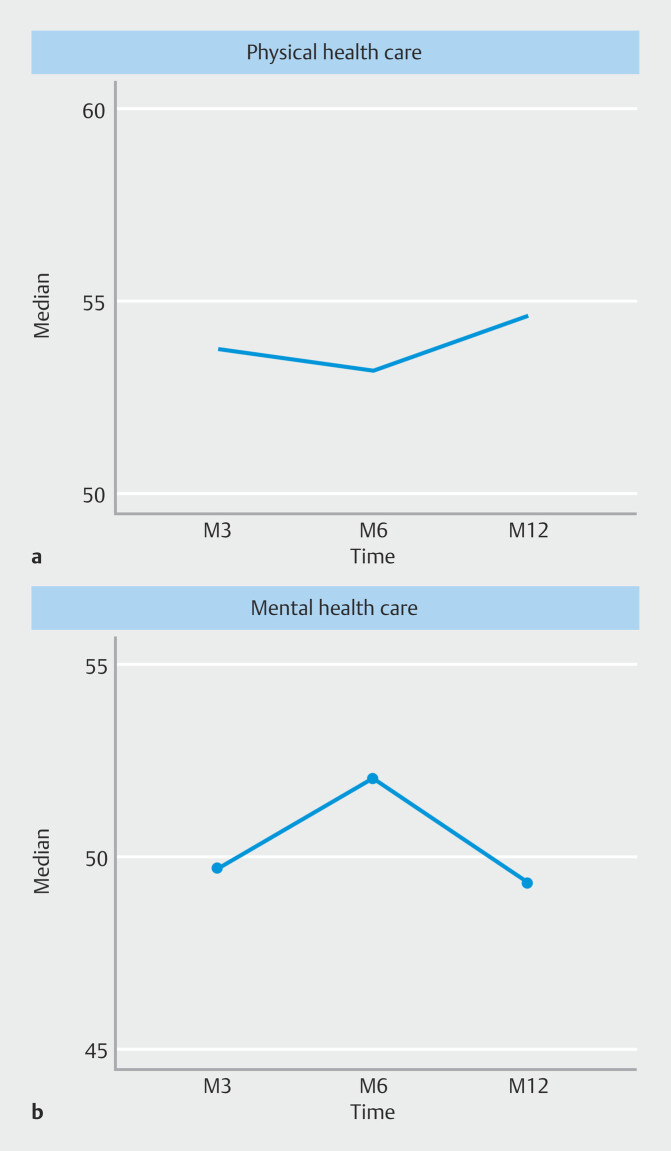
**a** Evolution of the SF12 physical health score. **b** Evolution of the SF12 mental health score.

QoL score (SF-12) was significantly improved for physical health ( Fig. 2 ). There was no improvement in SF-12 mental health score.

## Discussion

Obesity is a pandemic and the 5th leading cause of death worldwide 1 2 . Mortality has been shown to increase significantly above a BMI threshold ≥ 28 kg/m ^2^ 2 . Loss of life expectancy associated with obesity could be as high as 7.1 years for women and 5.8 years for men. Many related metabolic diseases such as type 2 diabetes, cardiovascular disease, steatohepatitis (MASH), and cancers are associated with obesity and are now targets of interventions to manage obesity (i.e., surgical, endoscopic, or pharmacological) 1 2 3 5 . Thus, bariatric endoscopy is referred to as EBMT. In this longitudinal prospective cohort study, patients with BMI ≥ 30 kg/m ^2^ and BMI < 35 kg/m ^2^ (Class I) with or without comorbidity or BMI ≥ 35 kg/m ^2^ and < 40 kg/m ^2^ (Class II) without comorbidity, which are complementary to surgical indications and required by National Health Insurance in France in order to get any further reimbursement, were included. In a previous French epidemiological study, prevalence of Class I obesity was 11.9% and Class II obesity was 3.1%, with total obesity prevalence of 17%.

ESG is one of the most commonly used endoscopic tools in management of obesity, along with IGB 5 . Recently, international guidelines from ASGE and ESGE have suggested use of ESG in conjunction with lifestyle management in obese patients with BMI ≥ 30 kg/m ^2^ 5 . In many studies, ESG has been shown to induce significant weight loss at 12 months, with TBWL ranging from 5% to 15% 5 6 7 8 9 10 11 12 13 14 15 . The strength of evidence-based medicine has recently increased with publication of two RCTs: the MERIT trial using the Apollo Overstitch device (Boston Scientific Corporation, Marlborough, Massachusetts, United States) and the European trial from Huberty et al. using the Endomina device (Endo Tools Therapeutics S.A., Gosselies, Belgium) 6 7 . To date, no comparative study has examined outcomes with the two devices on the market, but results in the literature and these two RCTs appear very conclusive 5 6 7 9 . The MERIT study was a double-blind, multicenter RCT evaluating safety and efficacy of ESG plus LSM in patients with Class I and Class II obesity 7 . A total of 209 patients were enrolled and, at 12 months, EWL was significant (49.2% vs 3.2%; *P* < 0.0001) and TBWL was also significant (13.6% vs 0.6%; *P* = 0.001). There was a significant reduction in comorbidities. AEs occurred in 2% of patients. The European RCT from Huberty et al. included 71 patients with a mean BMI of 34.6 kg/m ^2^ and was designed with a 6-month LSM crossover 6 . EWL and TBWL were significantly improved compared with LSM alone (38.6% vs 13.4% and 11% vs 2.7%, respectively), and at 1 year, EWL ranged from 43.6% to 51.3% and TBWL from 10.9% to 11.5%. At 1 year, 75.6% were considered responders to LSM+ESG (TBWL > 5%). More recently, a multicenter study using the Endomina device in 67 patients followed for 12 months reported an EWL rate of 48.5% and a TBWL of 15.3%; QoL was also significantly improved, with no serious AEs 9 .

Results of this prospective study were comparable in 29 patients with a mean BMI of 34.08 kg/m ^2^ , including nine patients with Class II obesity. At 1-year follow-up after 6 months of LSM and ESG, EWL reached a rate of 40.98% ( *P* < 0.001); TBWL reached 11.05% (p<0.001), with 81.2% being responders (TBWL > 5%). Nadir of these results was reached at 6 months. Regarding BMI, there was a significant decrease from 34.06 to 30.52 ( *P* < 0.001) and, at 1 year, there were no patients with Class II obesity and only 16 with Class I obesity; the remaining patients were reclassified as overweight ( *P* < 0.001).

In this study, improvement in glycemic control was observed with significant reductions in both glycemia and HbA1c. There were significant decreases in ALAT and GGT, which may be related to improvements in MASH. In addition, systolic arterial pressure was significantly reduced after 6 months and diastolic arterial pressure was significantly reduced after 1 year. Few studies have investigated metabolic effects of ESG. A recent systematic review and meta-analysis found only four studies with metabolic assessment (480 patients) among 35 relevant studies with a total of 7,525 patients 16 . TBWL at mid-term follow-up in this study was 15.4%; resolution of diabetes occurred in 55.4%, resolution of arterial hypertension in 62.8%, and resolution of dyslipidemia in 56.3%. The authors also looked at obstructive sleep apnea, which resolved in 51.7% of cases, but we did not investigate sleep apnea in our study. Therefore, the aim of ESG cannot be limited to weight loss outcomes but also includes metabolic effects.

In this prospective study, only one patient experienced an AE, which was epigastric pain (3.4%), resulting in a 2-day prolongation of hospital stay (AGREE II). In the literature, the rate of AEs ranges from 0.5% to 2.26% 5 6 7 8 9 10 11 12 13 14 15 17 . In addition, this study evaluated risk of nutritional or biological deficiencies, which has not been previously evaluated. No significant nutritional deficiency was found at 12 months for anemia, prealbumin, vitamin D, vitamin A, vitamin B9, or vitamin B12. To our knowledge, nutritional deficiency assessed by serum measurements of vitamins B12, B1, B9, A, C, D, K, E, iron, and ferritin has only been investigated in one study with a series of 20 patients, which did not demonstrate any abnormality 18 .

This study also included an assessment of QoL using the SF-12 questionnaire (mental and physical). This was repeated at each examination at 1, 3, 6, and 12 months. QoL (SF-12) was significantly improved for physical health. There was a trend toward improvement in SF-12 mental health at 12 months.

## Conclusions

In conclusion, ESG associated with LSM in patients with Class I obesity, with or without comorbidity, or Class II obesity without comorbidity, followed for 1 year, results in significant TBWL (> 5%) and EWL (> 25%) improvement, in accordance with the primary objective of this series. A significant decrease in BMI was also observed. In addition, QoL was significantly improved after 1 year. Metabolic improvement was also shown for glucose control, liver function tests, and arterial pressure. No biological deficiency occurred during follow-up and the morbidity rate was very low.
